# Longitudinal Network Changes and Phenoconversion Risk in Isolated REM Sleep Behavior Disorder

**DOI:** 10.21203/rs.3.rs-4427198/v1

**Published:** 2024-05-28

**Authors:** David Eidelberg, Chris Tang, Yoshikazu Nakano, An Vo, Nha Nguyen, Katharina Schindlbeck, Kathleen Poston, Jean-François Gagnon, Ronald Postuma, Martin Niethammer, Yilong Ma, Shichun Peng, Vijay Dhawan

**Affiliations:** The Feinstein Institutes for Medical Research; The Feinstein Institutes for Medical Research; The Feinstein Institutes for Medical Research; The Feinstein Institutes for Medical Research; Albert Einstein College of Medicine; The Feinstein Institutes for Medical Research; Stanford University; Université du Québec à Montréal; McGill University; The Feinstein Institutes for Medical Research; Center for Neurosciences, Institute of Molecular Medicine, The Feinstein Institutes for Medical Research, Manhasset, New York, USA.; Center for Neurosciences, Institute of Molecular Medicine, The Feinstein Institutes for Medical Research, Manhasset, New York, USA.; The Feinstein Institutes for Medical Research

## Abstract

Isolated rapid eye movement sleep behavior disorder (iRBD) is a prodromal syndrome for Parkinson’s disease (PD) and related *α*-synucleinopathies. We conducted a longitudinal imaging study of network changes in iRBD and their relationship to phenoconversion. Expression levels for the PD-related motor and cognitive networks (PDRP and PDCP) were measured at baseline, 2 and 4 years, along with dopamine transporter (DAT) binding.

PDRP and PDCP expression increased over time, with higher values in the former network. While abnormal functional connections were identified initially within the PDRP, others bridging the two networks appeared later. A model based on the rates of PDRP progression and putamen dopamine loss predicted phenoconversion within 1.2 years in individuals with iRBD. In aggregate, the data suggest that maladaptive reorganization of brain networks takes place in iRBD years before phenoconversion. Network expression and DAT binding measures can be used together to assess phenoconversion risk in these individuals.

## Introduction

Isolated rapid eye movement (REM) sleep behavior disorder (iRBD) is a parasomnia that is characterized by loss of muscle atonia and abnormal behaviors occurring during REM sleep. It is considered a prodromal stage of *α*-synucleinopathies, which include the major Lewy body disorders, i.e., Parkinson’s disease (PD) and dementia with Lewy bodies (DLB)^1,2^. Indeed, 60% of iRBD patients develop overt clinical signs of these disorders over 10 years^3,4^. Nevertheless, predicting when an individual with iRBD will develop signs and symptoms of PD or DLB is a major challenge. At a broader level, iRBD provides a unique population in which to assess the efficacy of potential disease-modifying therapies in at-risk individuals. That said, accurate determination of disease progression in these individuals is a daunting task given that characteristic clinical manifestations have yet to appear.

In a previous study with [^18^F]-fluorodeoxyglucose (FDG) positron emission tomography (PET), we found that iRBD is associated with elevated expression of the PD-related motor network (termed PD-related metabolic pattern, PDRP)^5–7^. Likewise, reduced dopamine transporter (DAT) binding in the putamen, reflecting presynaptic dopaminergic attrition, has been documented in iRBD and proposed as a potential predictor of phenoconversion^4,8^. In the current longitudinal PET study, we used FDG PET to examine the time course of PDRP expression in iRBD patients. Given that cognitive impairment has been found to be a risk factor for phenoconversion in iRBD, we also evaluated parallel changes in the expression of the PD-related cognitive network (termed PD-related cognition pattern, PDCP)^5,7,9^ in the same individuals. In addition to measuring longitudinal changes in PDRP and PDCP expression in unconverted iRBD subjects, we employed advanced computational techniques to study the gain and loss of specific metabolic connections linking nodes within and between the two disease topographies^10^, and assess the impact of the connectivity changes on network structure and function. Finally, we used [^18^F]-fluoropropyl-β-CIT (FPCIT) PET to determine the relationship of the network changes to loss of caudate and putamen DAT binding in iRBD, and the complementary role of these measures as predictors of phenoconversion.

## Results

### Longitudinal PD network changes in iRBD

#### Changes in network expression levels over time

**Figure 1:**
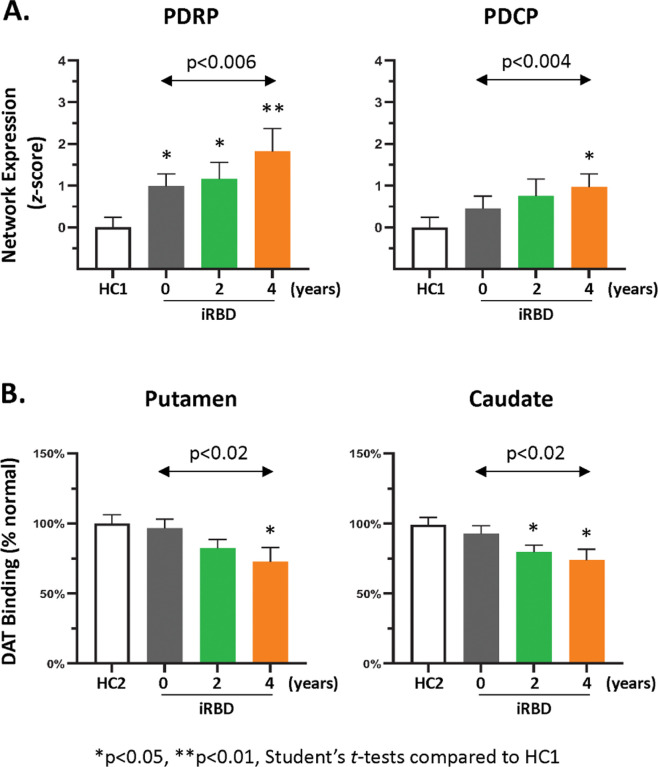
Longitudinal changes in metabolic network expression and dopamine transporter (DAT) binding. **(A)** Expression values (mean±SE) for the PDRP (*left*) and PDCP (*right*) are displayed for the iRBD cohort at baseline, 2 years, and 4 years and compared with healthy control (HC1) values (see text). Significant increases are observed over time for both networks. [Network expression at each timepoint was z-scored with respect to corresponding HC1 values.] **(B)**DAT binding (mean±SE) in the putamen (*left*) and caudate (*right*) are displayed for the iRBD subjects at each timepoint and compared to healthy control (HC2) subjects (see text). Significant declines were observed over time in both regions. [DAT binding at each timepoint was expressed as the percentage of the healthy control (HC2) mean for each region. Changes over time are represented by horizontal arrows. Differences from healthy control values at each are represented by asterisks (*p<0.05, **p<0.01).]

PDRP expression (Fig. 1A, *left*) was elevated in the iRBD cohort at all three timepoints relative to healthy control (HC1) subjects (baseline: p < 0.02, 2 years: p < 0.02, and 4 years: p < 0.002); elevations in PDCP expression (Fig. 1A, *right*) reached significance only at 4 years (p < 0.03; Student’s *t-tests*). Nonetheless, significant increases over time were observed for both networks (PDRP: F_(2,34)_ = 6.70, p < 0.006; PDCP: F_(2,34)_ = 7.83, p < 0.004; one-way RMANOVA), with greater expression at 4 years compared to baseline (PDRP: p < 0.005; PDCP: p < 0.003; post-hoc paired Bonferroni tests). Accordingly, estimated rates of progression were similar for the two networks (PDRP: 0.23 points/year p < 0.007, PDCP: 0.21 points/year p < 0.005; IGM). Longitudinal changes in PDRP and PDCP expression were closely related in the iRBD cohort (r = 0.89, p < 0,0001; Bland-Altman within-subject correlation).

#### Nodal analysis

Analysis of regional metabolic activity at key PDRP nodes (**Fig. S1A-D**) revealed significant baseline elevations in iRBD in the posterior putamen/globus pallidus (GP) (p < 0.02) and cerebellar vermis (p < 0.02 compared to HC1; Student’s *t*-test). At baseline, regional metabolism was marginally elevated in the pons (p = 0.09), but no change was present in the ventral lateral thalamus (p = 0.16). That said, significant elevations relative to normal were observed in all four of these nodes by the final timepoint (p < 0.02; Student’s *t*-tests). Indeed, metabolic activity increased over time in each of these regions (posterior putamen/GP: F_(2,34)_ = 5.52, p < 0.02; cerebellar vermis: F_(2,34)_ = 5.22, p < 0.02; dorsal pons: F_(2,34)_ = 8.26, p < 0.003; ventrolateral thalamus: F_(2,34)_ = 8.48, p < 0.003; one-way RMANOVA). In contrast to the major subcortical PDRP nodes, local metabolic activity did not change over time in sensorimotor, premotor, and parieto-occipital PDRP regions (p > 0.24; one-way RMANOVA), nor did these values differ from normal at any of the longitudinal timepoints (p > 0.13).

Nodal changes in the PDCP space were comparatively less striking. Significant declines in local metabolic activity were seen in the inferior parietal lobule (F_(2,34)_ = 7.23, p < 0.004; one-way RMANOVA), but deviations from normal in this region were Significant only at the third timepoint (p < 0.05 compared to HC1; Student’s *t*-test). That said, metabolic activity at the other major PDCP nodes did not change over time (p > 0.25; one-way RMANOVAs); reductions in these regions did not reach significance even at the last imaging timepoint (p > 0.08).

### Changes in metabolic connectivity and network organization in iRBD

#### Gain and loss of functional connections

**Figure 2:**
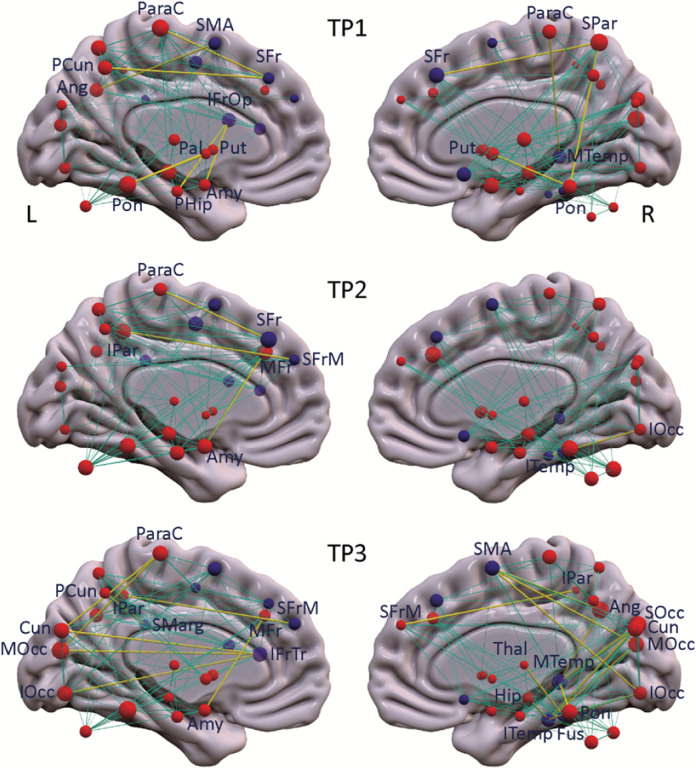
Metabolic connections gained at each timepoint in the iRBD cohort. PDRP and PDCP nodes (represented respectively by red and purple spheres) are displayed, with the radius of each node proportional to the degree centrality (number of connections) at each timepoint (TP). Significant connections gained at each timepoint compared to healthy subjects (**Tables S1A-C**) are represented by yellow lines, with thickness proportional to the strength of the connections (see Methods). Normal connections between network nodes are represented by cyan lines.

To identify the altered regional interactions between PD network nodes that underlie the differences in network metrics (see Methods), we evaluated the gain and loss of metabolic connectivity at each timepoint with respect to the HC1 subjects. The specific connections gained in the iRBD group, i.e., those present in iRBD but absent in healthy subjects, for the three timepoints are listed in **Table S1A**, *top* and displayed in Fig. 2. At the earliest iRBD timepoint, we noted Significant gain of connections in the PDRP space, i.e., PDRP–PDRP, preferentially linking metabolically active nodes located in the core zone of the network (**Table S1A**, *red font*). At later timepoints, the connections gained tended to link metabolically active core nodes with their underactive counterparts in the network periphery (**Table S1A**, *blue font*). These connections involved mainly those linking the supplementary motor area (SMA) with posterior cerebral cortical regions, and the amygdala with the middle frontal gyrus (BA 46). These connections were evident only at the final timepoint or in the case of the amygdala–prefrontal connection, at both the second and third timepoints.

The PDRP–PDRP connections that were lost, i.e., those that were present in healthy subjects but absent in iRBD are listed in **Table S1A**, *bottom*. At early timepoints (first timepoint alone or in the first two timepoints), loss of normal functional connections involved mainly those linking metabolically active nodes such as the putamen and cerebellum, and linking active and underactive nodes in the cerebellar vermis and middle frontal gyrus (BA 46), respectively. At later timepoints (last timepoint alone or in both the second and third timepoints), connectional losses were observed between metabolically active nodes in the thalamus and SMA, and between active and underactive nodes corresponding to the insula and the superior medial frontal gyrus (BA 9). Loss of connections between the metabolically active nodes, cerebellum and insula and between underactive nodes in the precuneus and inferior occipital gyrus (BA 19) were discerned at all three timepoints.

**Figure 3:**
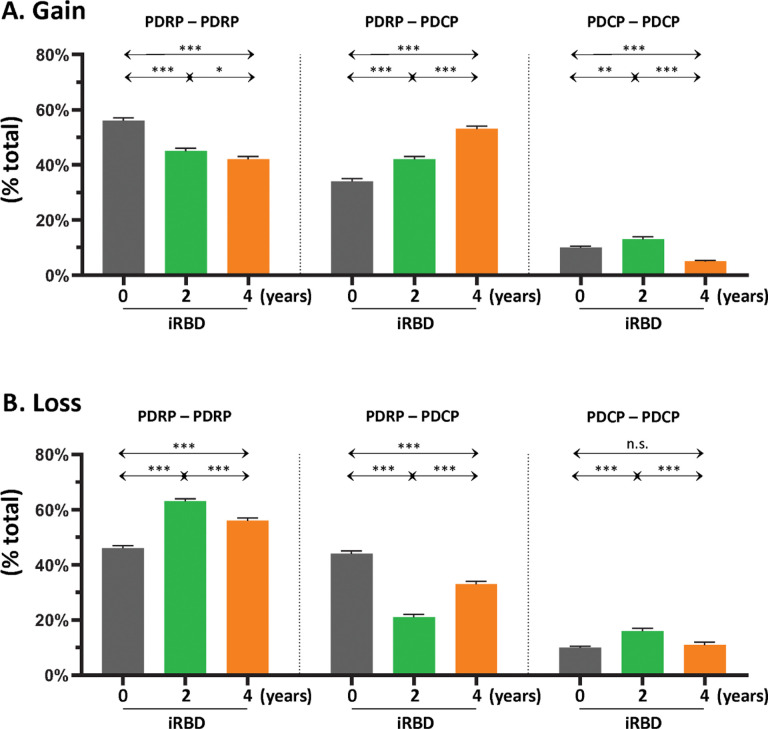
Gain and loss of functional connections within and between the PDRP and PDCP networks. **(A)** *Left*: At baseline (*gray*), the majority of gained connections (see Methods) were between PDRP nodes, but the proportion (% total) of PDRP–PDRP connections declined incrementally at the 2-year (*green*) and 4-year (*orange*) timepoints. *Middle*: Over the same time period, stepwise increases in gained connections were observed between PDRP and PDCP nodes (PDRP–PDCP). *Right*: By contrast, proportionally fewer connections were gained between PDCP nodes (PDCP–PDCP), with declines in this category of connections over time. **(B)** The percentage of healthy connections that were lost at baseline (see Methods) was similar for PDRP–PDRP (*left*) and PDRP–PDCP (*middle*) categories. Over time, however, the proportion of lost connections increased in the former category and declined in the latter. Loss of healthy PDCP–PDCP connections over time (*right*) was less than for the other connection categories, with no Significant change over time.

From a quantitative standpoint, the gain in intrinsic connections across the whole PDRP space (Fig. 3A, *left*) was 56% at the initial timepoint, which declined to 42–45% at the second and third timepoints. This contrasted with the stepwise increases in the gained connections that were observed linking PDRP and PDCP nodes (Fig. 3A, *middle*; **Table S1B**, *top*), which ranged from 34% at the first timepoint to 53% at the last timepoint, and with the low-level gain of intrinsic PDCP connections (< 13%) that was present at each of the three timepoints (Fig. 3A, *right*; **Table S1C**, *top*). Loss of connections was also noted, initially involving those linking PDRP nodes (Fig. 3B, *left*; **Table S1A**, *bottom*) or those linking PDRP with PDCP nodes (Fig. 3B, *middle*; **Table S1B**, *bottom*). At subsequent timepoints, loss increased in connections linking PDRP nodes, but was less pronounced for those linking PDRP with PDCP nodes. As with gain, loss in the PDCP space was minimal (< 11%) at all three timepoints (Fig. 3B, *right*; **Table S1C**, *bottom*). In aggregate, the findings suggest ongoing remodeling of PDRP functional connectivity beginning at the earliest prodromal stages of the disease process.

#### Changes in network metrics over time

**Figure 4:**
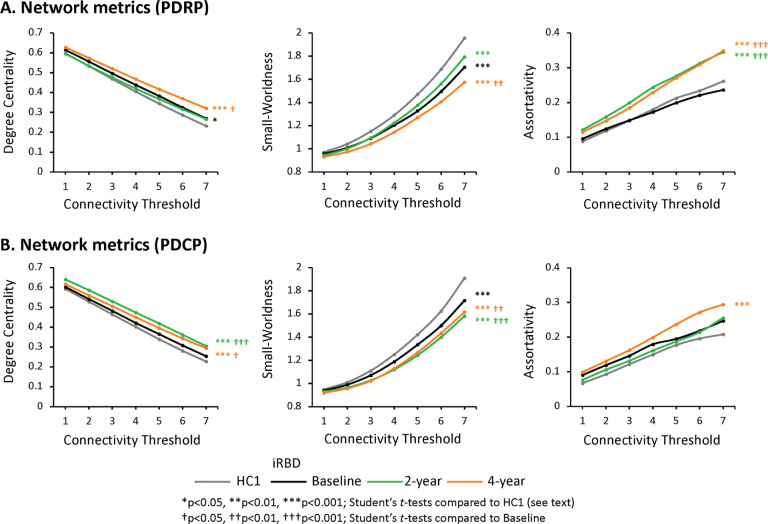
PDRP and PDCP network metrics at each timepoint. Relevant network metrics were plotted for **(A)**PDRP and **(B)** PDCP. For each network, degree centrality (*left*), small-worldness (*middle*), and assortativity (*right*) were plotted for the iRBD cohort at baseline (*black*), 2 years (*green*), and 4 years (*orange*); corresponding values in the healthy control (HC1) group are provided for reference (*gray*). [Group differences were evaluated using the general linear model across graph thresholds with Bonferroni corrections for multiple comparisons (see Methods). Post-hoc differences from HC1 are represented by asterisks (*p<0.05, **p<0.01, ***p<0.001); differences from baseline (timepoint 1) are represented by crosses (†p<0.05, ††p<0.01, †††p<0.001).]

To evaluate the changes in PD network structure and information flow that occur during the progression of iRBD, we computed key graph metrics for PDRP and PDCP at each timepoint (see Methods). For PDRP, we found that degree centrality (Fig. 4A, *left*) was marginally elevated at the first timepoint (P_**CORR**_=0.034) but not the second; Significant increases in this metric were observed, however, at the final timepoint (P_**CORR**_<0.001). Significant reduction in the clustering coefficient was noted at the first and third timepoints (P_**CORR**_<0.0001). The characteristic path length was elevated at all three timepoints (P_**CORR**_<0.001); the largest values for this metric were observed at the last timepoint. Accordingly, abnormal reductions in PDRP small-worldness (Fig. 4A, *middle*) were present at all three iRBD timepoints (P_**CORR**_<0.0001), with the smallest values at the last timepoint. This suggests a progressive imbalance in information flow through the PDRP network, which begins early in the prodromal period. We additionally measured the assortativity coefficient (Fig. 4A, *right*), an index of the homogeneity of connections in the network space (see Methods). While this graph metric was normal at the initial iRBD timepoint, values were significantly elevated at the latter two timepoints (P_**CORR**_<0.0001). Thus, while reduction in PDRP small-worldness is evident early in the course of iRBD, other maladaptive features, notably increases in network assortativity, develop later in the prodromal period.

For PDCP, similar changes were observed over time for the various graph metrics. At baseline, degree centrality (Fig. 4B, *left*) was normal in the network space, but Significant elevations in this metric were present at the second and third timepoints (P_**CORR**_<0.001). That said, Significant reductions in the clustering coefficient, increases in characteristic path length (P_**CORR**_<0.0001), and consequently, reduced small-worldness (P_**CORR**_<0.0001; Fig. 4B, *middle*) were present at all three timepoints. These abnormalities were largest at the final timepoint. In contrast to PDRP, assortativity (Fig. 4B, *right*) did not differ significantly from normal at either the first or second timepoints. This metric was elevated, however, at the last timepoint (P_**CORR**_<0.0001).

#### Longitudinal clinical changes and dopaminergic time course

UPDRS motor ratings obtained at the time of imaging (baseline: 1.64 ± 1.69; 2 years: 3.00 ± 2.42; 4 years: 2.82 ± 2.27 (mean ± SD)) did not change significantly over time (F_(2,34)_ = 1.15, p = 0.34; one-way RMANOVA). Significant changes in striatal DAT binding were noted, however, in this cohort. Putamen DAT binding (Fig. 1B, *left*) declined over time (F_(2,27)_ = 6.15, p < 0.02; one-way RMANOVA); values were lower values at 4 years compared to baseline (p < 0.02; post-hoc paired Bonferroni test) and to healthy control (HC2) subjects (p < 0.03; Student’s *t-test*). The mean rate of decline in putamen DAT binding was estimated to be −4.8%/year (p < 0.002; individual growth model (IGM)). Caudate DAT binding (Fig. 1B, *right*) also declined over time in the iRBD cohort (F_(2,27)_ = 6.22, p < 0.02; one-way RMANOVA); values were lower at 4 years relative to baseline (p < 0.02; post-hoc Bonferroni test), and at 2 years and 4 years compared to healthy subjects (p < 0.02; Student’s *t-tests*). The rate of decline in caudate DAT binding in iRBD was estimated to be −4.4%/year (p < 0.0007; IGM), which did not differ from the corresponding rate estimate for the putamen (p = 0.49; region × time interaction effect). Significant within-subject correlations were not evident for longitudinal changes in UPDRS motor ratings with concurrent increases in PDRP or PDCP expression (p > 0.11) or with declines in caudate or putamen DAT binding (p > 0.48; Bland-Altman correlations). Correlations between changes caudate/putamen DAT binding and PDRP expression were of borderline significance (p = 0.08 for both regions); those with changes in PDCP expression were not Significant (p > 0.13; Bland-Altman correlations).

### Prediction of time of phenoconversion in individuals with iRBD

#### Predictions based on baseline PDRP expression

**Figure 5:**
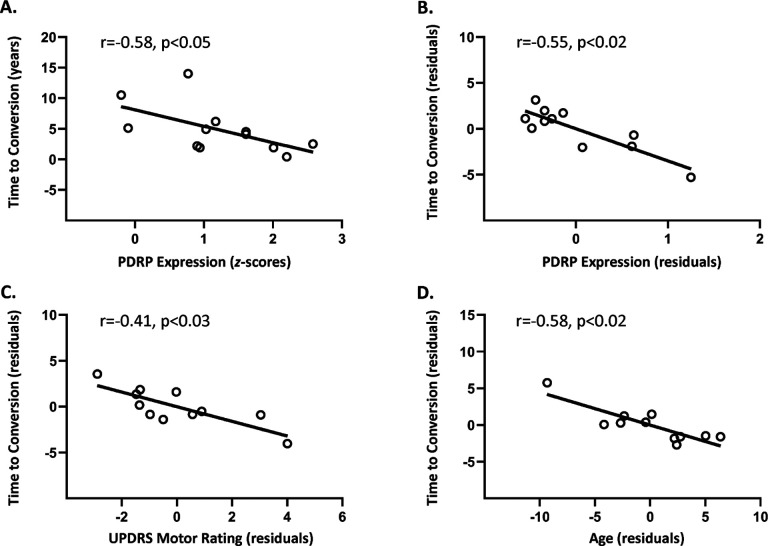
Correlation with the time from imaging to phenoconversion. **(A)** The time to phenoconversion (years) from the imaging study correlated inversely (r=−0.58, p<0.05; Pearson correlation) with PDRP expression measured at baseline in the 12 iRBD subjects from the cross-sectional cohort who subsequently phenoconverted to PD/DLB (see text). **(B)** Leverage plot analysis further illustrates the Significant inverse correlation of the time to phenoconversion with PDRP expression after controlling for age, sex, UPDRS motor ratings, and iRBD duration (r=−0.55, p<0.02; partial correlation). **(C, D)** Leverage plots show additional correlations of the time to phenoconversion with baseline UPDRS motor ratings (r=−0.41, p<0.03) and age (r=−0.58, p<0.02; partial correlations). Correlations with sex and iRBD duration were not Significant (p>0.5).

We computed baseline PDRP and PDCP expression in [^99m^Tc]-ethylcysteinate dimer (ECD) single photon emission computerized tomography (SPECT) perfusion scans obtained in 17 iRBD subjects as previously reported (see Methods). For both networks, expression values were abnormally elevated in the iRBD group (PDRP: p < 0.004; PDCP: p < 0.03 compared to HC3; Student’s *t-tests*). Of the iRBD subjects, 12 subsequently developed signs of PD (n = 8) or DLB (n = 4) an average of 4.8 ± 3.9 years (range: 0.4 to 14.0 years) after imaging. A Significant inverse correlation (r=−0.58, p < 0.05; Pearson correlation) was seen between baseline PDRP expression and the time to conversion (Fig. 5A). An analogous correlation was not observed with baseline PDCP values (r = 0.32, p = 0.32). Furthermore, PDRP expression, along with covariates of age, sex, iRBD duration, and UPDRS motor ratings were collectively entered into a multiple regression model to predict the time to conversion for the 12 phenoconverters (see Methods). Indeed, the relationship between PDRP expression and time to conversion remained Significant (p < 0.02; partial correlation) after adjusting for the other covariates (Fig. 5B, leverage plot). While time to conversion also correlated with UPDRS motor ratings (p < 0.03) and age (p < 0.02) (Fig. 5C, D), those with sex and iRBD duration were not Significant (p > 0.5). Indeed, of the 10 iRBD subjects in this cohort who converted to PD or DLB in the seven years after imaging, nine had baseline PDRP scores greater than 0.9. Of the five non-converters, only two had values in this range. These findings suggest that people with iRBD who have high PDRP levels have a greater risk of subsequent phenoconversion than their low expression counterparts.

#### Predictions based on longitudinal imaging data

**Figure 6:**
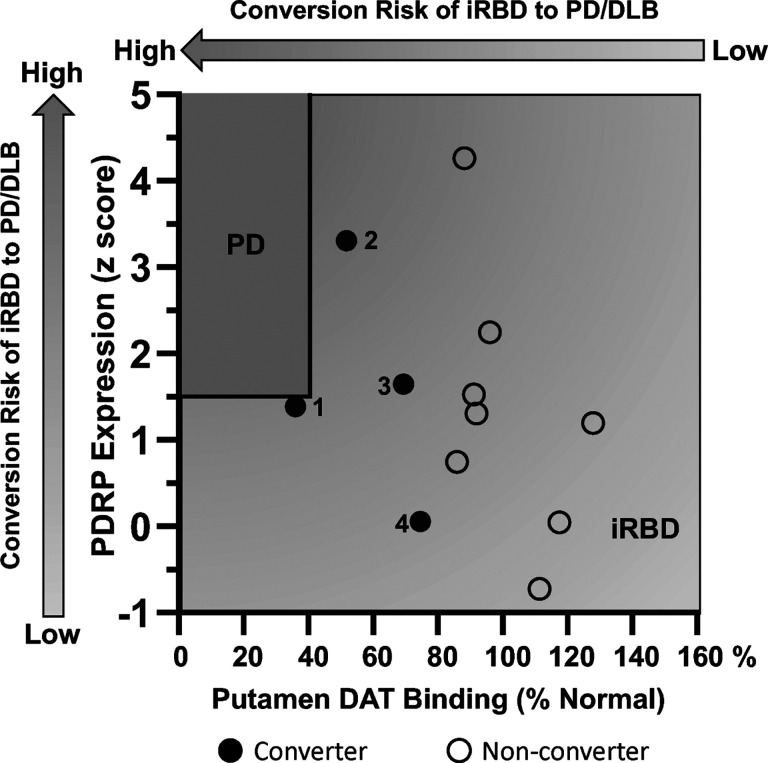
Phenoconversion risk in iRBD subjects. PDRP expression values (*y-axis*) were plotted against putamen DAT binding measurements (*x-axis*) recorded at the final imaging timepoint for each of the longitudinal iRBD subjects. The four iRBD subjects who converted to PD (Subjects #1–3) or DLB (Subject #4) within 7 years after the final imaging timepoint are represented by black circles (see text). The remaining iRBD participants who did not phenoconvert during the observational phase of the study are represented by open circles. Shaded arrow bars indicate the hypothetical risk of phenoconversion ranging from low to high, associated with higher PDRP expression (*left side vertical bar)* and lower putamen DAT binding (*top horizontal bar*) in iRBD subjects (see text). [The “PD zone” (*top left*) was defined based on historical data of early-stage PD patients, for whom PDRP expression was typically greater than +1.5 and putamen DAT binding was below 40% of normal mean (*solid black lines*) (see text).]

Over the course of the longitudinal study, 4 of the 13 iRBD participants in the current longitudinal PET cohort (Fig. 6, *black circles*) converted to PD (Subjects #1–3) or DLB (Subject #4) at 0.1, 3.6, 5.5, and 6.4 years (mean 3.90 years) respectively after the final imaging timepoint. Based on our prior imaging studies of early PD^11,12^, we found that PDRP expression was elevated above + 1.5 at the time of clinical diagnosis, whereas putamen DAT binding was reduced below 40% of the normal mean value. Using the progression rates for these measures estimated by IGM in the iRBD longitudinal cohort (see Methods), we calculated the time it would take to cross *both* PD thresholds beginning at the individual’s final pre-conversion scan. For the four converters, the model predicted times to conversion of 0.5, 2.5, 6.2, and 6.2 years (mean 3.85 years). These estimates were in close agreement with the observed phenoconversion times, with error ranging from − 1.2 to + 0.7 years. Thus, the data suggest that the risk of pheonoconversion in individual iRBD subjects can be accurately estimated using a combination of imaging measures. Individuals with high PDRP levels and low putamen DAT binding are at relatively greater risk. This is evident in the four converters in whom values were nearer to the predefined “PD zone” (Fig. 6, *top left, dark gray rectangle*). Of the non-converters (*open* circles), three subjects had normal values for both measures (*bottom right*) and were furthest away from the “PD zone.” These individuals were considered to be at least risk. The remaining iRBD non-converters had abnormal values for one or the other measure but not both and were considered to be at intermediate risk.

## Discussion

In this longitudinal study, we observed progressive increases in PDRP and PDCP expression in iRBD, as well as concurrent declines in caudate and putamen DAT binding. While in the iRBD cohort, progression rates were similar for the two PD networks, expression values for the PDRP were greater than PDCP at each timepoint, a finding consistent with earlier cross-sectional studies^6^. Compared to healthy control subjects, PDRP expression was already elevated at baseline, whereas PDCP values did not reach abnormal levels until the final imaging timepoint. Likewise, the rate of decline in DAT binding was similar for the caudate and putamen, with relative preservation of nigral dopaminergic input to both structures initially, followed by modest reductions to 80–85% of the normal mean at the final timepoint. Importantly, four iRBD participants converted to PD/DLB during the subsequent 7 years of clinical follow-up. Thus, our imaging data were mainly shaped by the evolution of distinct PD network topographies during the prodromal phase of the illness. Indeed, network progression in iRBD was accompanied by the formation of abnormal functional connections linking PDRP nodes to one another and to PDCP nodes. These connectivity changes are likely maladaptive as suggested by the appearance of abnormal network configurations soon after the diagnosis of iRBD.

In PD, increases in PDRP expression have been attributed mainly to changes in cortico-striato-pallido-thalamo-cortical motor circuits taking place as a downstream consequence of nigrostriatal dopaminergic degeneration^7,13,14^. That said, the abnormal network elevations in iRBD may also be influenced by changes involving monoaminergic and/or cholinergic pathways in the brainstem and basal forebrain. Indeed, measurements of local metabolic activity at key network nodes (**Fig. S1**) suggest that while the earliest regional abnormalities were localized to basal ganglia and cerebellum, PDRP core regions in the ventral thalamus and dorsal pons reach significance only the final timepoint. While longitudinal metabolic changes in cortical PDRP regions were not identified, Significant declines were observed in the inferior parietal PDCP node. It is unclear, however, whether this change was the result of loss of cholinergic afferents to the region or α-synuclein deposition, or both. In the absence of rigorous neuropsychological testing, it is also unclear how metabolic changes in this region relate to concurrent cognitive performance in individuals with iRBD.

That said, the results of the connectivity analysis generally accord with the α-synuclein propagation hypothesis^15^. In early iRBD, gain and loss of connections were observed in the PDRP space, involving mainly those linking the basal ganglia to the brainstem and cerebellum. We note that the initial gain of functional connections was between metabolically active core nodes, with subsequent development of abnormal connections linking core nodes to their less active counterparts in the network periphery. Indeed, a similar pattern of connectional gain was previously reported in a cross-sectional analysis of connectivity changes in PD patients^10^. On the other hand, loss of the normal core-to-core and core-to-periphery connections was observed both early and later on in the course of iRBD. For example, we noted early loss of normal connections linking the cerebellum and contralateral putamen in iRBD, suggesting that this pathway is likely disrupted years before the onset of parkinsonian symptoms. Other potentially relevant connections, such as those linking the thalamus to the paracentral lobule (which includes the SMA) and the amygdala to the parahippocampal gyrus, are lost later in the course of iRBD. How these changes related to the post-conversion phenotype of individuals with iRBD remains to be elucidated. When considered in aggregate, however, the findings point to dynamic network remodeling in iRBD, beginning in the PDRP core zone and spreading outward over time into the periphery.

Interestingly, during iRBD progression, the abnormal connections that were gained were not limited to those between PDRP nodes. Indeed, a Significant stepwise increase in abnormal connections linking PDRP to PDCP nodes was noted over time (Fig. 2; Fig. 3A, *middle*). These connections were predominantly cortico-cortical, initially linking frontal lobe regions, with the subsequent appearance of abnormal connections linking the amygdala and parahippocampal gyrus to the frontal operculum. Over time, abnormal connections become evident linking frontal, temporal, and parietal regions with occipital association cortex. That said, gain and loss of functional connections linking PDCP nodes was of small magnitude compared to those between PDRP nodes or between PDRP and PDCP nodes.

We additionally found that the gain and loss of individual connections likely had a detrimental influence on the structure and function of these networks. In this regard, we found evidence of reduced small-worldness for both PDRP and PDCP, beginning at the first timepoint. This change is attributed to the abnormal reductions in connections between nearest neighbors (low clustering coefficient) that were evident in both networks, as well as increases in the average number of edges separating individual nodes from the others (high characteristic path length). These topological changes suggest an imbalance between regional specialization (segregation) and parallel processing (integration), which may reduce the efficiency of information flow through both networks. It is also noteworthy that assortativity, the link-averaged correlation coefficient of degree centrality for pairs of connected nodes in a network^16–18^, is abnormally increased in iRBD beginning at the second timepoint for PDRP and the third timepoint for PDCP. This metric captures changes in connectional diversity that render networks more vulnerable to random attacks, fragmentation, and critical transitions^19–21^. We have previously described progressive increases in PDRP assortativity in multiple groups of PD patients^22^. The current data point to analogous network changes in iRBD, starting years before the onset of motor symptoms. Taken together, the decline in small-worldness and subsequent increase in assortativity seen in longitudinal iRBD are compatible with progressive compromise of information flow through PD-specific networks in prodromal stages of the disease.

In addition to the changes in PD network expression, we found that DAT binding declined over time in both the caudate and putamen, at similar rates for the two regions. In that regard, FPCIT PET provides complementary information concerning the evolution of dopaminergic deficits in iRBD and the relationship of these changes to the onset of parkinsonism^23^. While presynaptic dopaminergic deficits, particularly those localized to the putamen, are a recognized risk factor for phenoconversion^12,24^, these measurements, as well as other potential predictors such as severity of motor symptoms, autonomic dysfunction, and cerebrospinal fluid analysis, do not accurately foretell subsequent transition to clinically manifest PD^25,26^. That said, the longitudinal PET data presented in this study suggest that changes in PDRP expression and putamen DAT binding, when evaluated together, may predict phenoconversion more accurately than either measure alone. Indeed, in our earlier longitudinal PET studies of newly diagnosed PD patients^11,12^, we found that these individuals typically had PDRP expression values above + 1.5 and reductions in putamen DAT binding below 40% of the normal mean value^11,12,24^. Given estimates of the rate of change in these measures, we used the final pre-conversion scans of each iRBD participant to calculate the time needed to reach the prespecified cut-offs for early PD. While in close agreement with observed phenoconversion times determined independently by movement disorders experts, predictions made based on the model need to be replicated in prospective studies of larger iRBD cohorts.

In this regard, we note that only four of the 13 (31%) longitudinal iRBD subjects converted to PD/DLB over a total of 9.6 years (longitudinal imaging for an average of 4.3 years and follow-up clinical observation for an additional 5.3 years). The relatively small number of phenoconversions in this longitudinal cohort can be explained by the milder initial clinical features in these subjects compared to the 12 of 17 (71%) who phenoconverted in the cross-sectional study. Indeed, on enrollment, the phenoconverters in the former group had shorter mean iRBD duration than their cross-sectional counterparts (3.6 vs. 9.6 years), lower initial motor ratings (2.0 vs. 5.4 points), and longer time from initial imaging to phenoconversion (8.3 vs. 4.8 years). It is therefore likely that the iRBD participants in the longitudinal imaging study were further from phenoconversion at the time of enrollment than their counterparts in other studies.

It is noteworthy that other investigators have identified iRBD-related spatial covariance patterns (termed iRBDRPs) in FDG PET scans from iRBD subjects^27,28^, or from *de novo* PD patients with iRBD (termed PD-RBDRP)^29,30^. PD-RBDRP expression measured in iRBD subjects predicted future phenoconversion to PD in iRBD, although this pattern exhibited Significant topographical overlap with PDRP. More recently, a somewhat different multivariate mapping technique was applied to [^99m^Tc]-HMPAO SPECT cerebral perfusion scans acquired in iRBD and healthy volunteer subjects^31^. The resulting pattern was reported to be a good predictor of future phenoconversion in individuals with iRBD, although prospective validation of the marker is needed in independent pre-conversion samples. This approach contrasted with that employed in the current study in which we used the FDG PET-based PDRP, a highly reproducible network biomarker of PD, which was additionally validated in [^99m^Tc]-ECD SPECT perfusion scans^32,33^. This approach led to a Significant correlation between baseline PDRP expression and the time to phenoconversion, although further validation is desirable. It is also worth noting that new methods have recently been developed to measure PD network expression in individual subject scans. For example, dynamic [^18^F]-FPCIT PET can be used to quantify PDRP and PDCP expression in early phase perfusion scans, while caudate and putamen DAT binding can be measured in the late phase images^34^. Non-invasive measurements of network expression are also possible given that elevations of the resting-state fMRI-based PDRP have been documented in both PD and iRBD^5,35,36^. This approach may prove valuable as a non-invasive, widely accessible means of assessing the risk of phenoconversion in individuals with iRBD.

Given that metabolic pattern expression was found to be a sensitive measure of iRBD progression in our study, we performed a preliminary power/sample-size analysis using the PDRP expression as a progression biomarker in a hypothesized 2-year (2-timepoint), blinded disease-modification trial of individuals with iRBD randomized to active treatment versus placebo. The results show that randomizing 114–308 subjects may be sufficient to detect reductions in progression rate of 30–50% respectively with 80% power (two-sample Student’s *t*-test; G*Power 3.1). This suggests that PDRP measurements can enhance statistical power in such trials.

## Methods

### Subjects

We conducted a 4-year multi-center longitudinal imaging study of individuals with iRBD (13 men; age: 63.5 ± 8.4 years; baseline Unified Parkinson’s Disease Rating Scale (UPDRS) motor ratings: 1.5 ± 1.7 (mean ± SD)) who were recruited from the Sleep Disorders Clinics at The Feinstein Institutes for Medical Research (n = 8), University of Pennsylvania (n = 4), and Stanford University (n = 1). These participants were scanned at baseline (1.7 ± 2.0 years from the diagnosis of iRBD) with [^18^F]-fluorodeoxyglucose (FDG) positron emission tomography (PET) to map cerebral glucose metabolism; scanning was repeated 2 years (n = 12) and 4 years (n = 10) later. For each of the subjects, longitudinal FDG PET scans were obtained locally at each site using the GE Advance tomograph at the Feinstein Institutes, Manhasset, NY (n = 8), the Siemens Biograph 40 mCT-S PET/CT at the Hospital of the University of Pennsylvania, Philadelphia, PA (n = 4), and the GE Advance tomograph at Stanford University, Palo Alto, CA (n = 1). Of the iRBD cohort, a subset additionally underwent longitudinal [^18^F]-fluoropropyl-β-CIT (FPCIT) PET at baseline (n = 13), 2 years (n = 10), and 4 years (n = 6) to measure dopamine transporter (DAT) binding in the caudate and putamen at each timepoint. These scans were performed within two months of FDG PET using the tomograph at the Feinstein Institutes. The details of this procedure are reported elsewhere^34^.

The following criteria were used for participant selection. *Inclusion*: (1) A diagnosis of iRBD by a sleep disorder specialist based on clinical history and polysomnographic confirmation; and (2) Minimum age of 30 years. *Exclusion*: (1) Known diagnosis of PD or other neurodegenerative disorder; (2) Unequivocal signs of parkinsonism on clinical examination and/or past or current histories of treatment with antiparkinsonian medications; (3) Prior history of stroke; (4) Use of neuroleptics, atypical antipsychotics, and antiemetics within the prior year; (5) Narcolepsy or other sleep disorders including moderate or severe obstructive sleep apnea; (6) Individuals receiving benzodiazepines, anticholinergics, selective serotonin reuptake inhibitors, tricyclic antidepressants, monoamine oxidase inhibitors, or other central nervous system active drugs were excluded if iRBD symptoms began after the introduction of these medications. If the iRBD symptoms predated the initiation of these medications, subjects were enrolled but medications were withheld for at least 12 hours before imaging. The participants were followed clinically for an additional 6.4 ± 2.0 years after the final imaging timepoint. During the clinical phase of the study, all subjects were evaluated for phenoconversion on an annual basis by movement disorders specialists at the participating sites. In this longitudinal iRBD cohort, four male participants (age: 68.9 ± 12.1 years; baseline UPDRS motor ratings: 2.0 ± 2.2; iRBD duration: 3.6 ± 2.8 years) converted to either PD (n = 3) or DLB (n = 1) at an average of 3.9 ± 2.8 years after the final imaging timepoint. Study protocols and consent forms were approved by the institutional review boards of the collaborating institutions. Written consent was obtained from each participant after detailed explanation of the procedures.

### Longitudinal image analysis

#### FDG PET:

Scans from the iRBD participants and a group of 17 age- and sex-matched healthy control subjects (HC1: 15 men and 2 women; age: 59.9 ± 10.7 years) were realigned, spatially normalized, and smoothed as described elsewhere^37^. Expression levels (subject scores) for the PDRP and PDCP networks were computed in each of the iRBD subjects and timepoints; the resulting values were standardized (z-scored) with respect to the HC1 group. These calculations were conducted using in-house ScAnVP (software freely available upon request at https://feinsteinneuroscience.org) as described elsewhere^7,38^.

In addition to the network calculations, we analyzed the regional data to assess the changes that occurred over time at key network nodes. This was done by measuring local metabolic activity in spherical volumes-of-interest (VOIs) (radius = 2–5 mm) centered on the peak voxel of each of the key regions^39,40^. The resulting values were then ratio-normalized by the global metabolic rate (GMR) for each scan and the changes over time in the iRBD group were determined for each of the regions.

#### FPCIT PET:

Scans from the subset of the iRBD cohort who were additionally scanned with FPCIT PET (see above) were analyzed at each timepoint as described elsewhere^34^. In each scan, standardized regions-of-interest (ROIs) were placed bilaterally on the caudate nucleus, putamen, and occipital cortex; scans were aligned to baseline such that identical ROI templates were applied to the analysis of the scans from all three timepoints^12^. At each timepoint, we estimated caudate and putamen DAT binding by the striatal-to-occipital ratio (SOR), defined as (striatum – occipital)/occipital counts in a single 10-min frame, beginning 90 min after tracer injection^12,34^. Averaged right and left DAT binding for the putamen and caudate nucleus were separately calculated for each subject and each timepoint, and compared with analogous values from a separate healthy control group (HC2: 4 men and 6 women; age: 60.0 ± 9.9 years).

At each iRBD timepoint, we compared the PDRP/PDCP scores and regional metabolism at key network nodes, as well as caudate/putamen DAT binding, with corresponding HC values using two-sample Student’s *t-tests*. Longitudinal changes in the imaging measures were assessed with one-way repeated measures analysis of variance (RMANOVA) corrected for multiple comparisons using paired Bonferroni tests. In addition, individual growth models (IGMs)^41,42^ were used to estimate progression rates for PDRP and PDCP expression over time in the iRBD longitudinal cohort, as well as for declines in caudate and putamen DAT binding in the same individuals. Relationship between changes in these measures over time were evaluated by computing Bland-Altman correlation coefficients. These analyses were performed using SAS Studio (SAS Institute, Cary, NC) software and considered Significant for p < 0.05, two-tailed.

### Changes in metabolic connectivity and network organization in iRBD

#### Gain and loss of specific connections

To identify functional connections between network nodes that are altered at each iRBD timepoint, we analyzed all Significant connection pairs using a method described previously for FDG PET^10,37,40^. In particular, we focused on connections linking: (a) PDRP nodes with other PDRP nodes, (b) PDCP nodes with other PDCP nodes, and (c) PDRP with PDCP nodes in the iRBD subjects at each timepoint, and compared the results to HC1. This approach allows for the identification of the connections gained in iRBD but absent in HC1 at each timepoint. Likewise, the loss of connections at each longitudinal timepoint was determined by those present in HC1 but absent in iRBD. To this end, we parcellated the brain into 95 ROIs based on the AAL atlas^43^, and used globally normalized metabolic activity in each region to construct matrices of pairwise correlations. For group-level analysis, we used bootstrap resampling (in-house Matlab script; MATLAB R2020a) to generate 100 samples for each group and timepoint. For each iteration, we computed pairwise nodal Pearson correlations. The median values of the iterates (100 bootstrap correlation estimates) were used to create an adjacency matrix for the network in each group and timepoint. These calculations were performed using the Machine Learning Toolbox in MATLAB R2020a.

By this scheme, the magnitude of the correlation (|*r*|) provided a measure of connectivity between network nodes for each network and timepoint. For a given pair of nodes, group differences in connectivity were described by the absolute difference (|*dr*|) in the two correlation coefficients^10,37^. For a connection to be gained in iRBD, we required that the magnitude of the correlation coefficient (|*r*|) that defined the associated graphical edge be greater than or equal to 0.6 (p < 0.05; Pearson correlation) in the iRBD group but not in HC, and that the corresponding absolute difference (|*dr*|) from HC be greater than 0.4. (The latter threshold was determined using the HC graph and permuting the regional labels 1000 times to create a set of pseudorandom correlations for each iteration as described elsewhere^10,37^. Differences exceeded chance for |*dr*|>0.4, p < 0.05; permutation test.) By the same token, the loss of a normal connection was considered Significant if |*r*| for the particular graphical edge was greater than or equal to 0.6 in HC but not in the iRBD group, and that the corresponding absolute difference from HC was greater than 0.4. For each Significant edge, the connections that satisfied these criteria were confirmed by bootstrap resampling (100 iterations) using the Statistics and Machine Learning Toolbox in MATLAB R2020a. The resulting metabolic connections linking pairs of PD network nodes were considered for further analysis if they: (1) lay along a known anatomical pathway; and (2) were separated by no more than two sequential hops along the graph^10,44^.

Gain and loss of connections was quantified by the percentage of the total at each iRBD timepoint that linked nodes belonging to one network (PDRP–PDRP, PDCP–PDCP) or that bridged the two networks (PDRP–PDCP). Group differences in the gain and loss of connections relative to HC1 were evaluated using one-way measures analysis of variance (ANOVA) with post-hoc Bonferroni tests. These analyses were performed using MATLAB R2020a. Results were considered Significant for p < 0.05, corrected for multiple comparisons.

#### Changes in network architecture over time

Apart from longitudinal changes in PDRP/PDCP progression in the iRBD cohort, we evaluated several topological features that influence information flow through the network. To this end, we computed the following metrics on weighted undirected graphical links and compared the measures at each timepoint with analogous values for the HC1 group as detailed elsewhere^10,22,37^.

1. *Degree centrality*: the number of connections linking network nodes, divided by the total number of nodes that constitute the network. This measure reflects overall connectivity within the network space.
2. *Clustering coefficient*: the likelihood that the nearest neighbors of a node will also be connected. This metric provides an index of the degree that groups of nodes are clustered together in a given network.
3. *Characteristic path length*: the shortest path length between two nodes averaged over all pairs of nodes within the network space^16,45^. This metric represents the average number of hops needed to connect a given node to the others, which is inversely related to the efficiency of information flow across the entire network.
4. *Small-worldness*: the ratio of clustering coefficient to characteristic path length, normalized to the corresponding values from an equivalent random graph^46^. This measure quantifies the ratio of regional specialization (segregation) to parallel processing (integration) of information flow along the network.
5. *Assortativity coefficient*: the correlation coefficient between the degree centrality for the nodes on opposite ends of a link, averaged across a given network^16–18,20^. This measure represents the propensity for nodes to form connections with nodes having similar or different attributes. As a general measure of connectional heterogeneity, assortativity provides a measure of overall network stability^19,22,47,48^.

These metrics were computed using the Brain Connectivity Toolbox^45^ and an in-house Matlab script (MATLAB R2020a). The results were plotted over a range of connectivity thresholds (r = 0.3 to 0.6, at 0.05 increments, corresponding to graph densities between 25% and 60%)^10,22,37^ to demonstrate the consistency of group differences in a given metric over multiple adjacent levels. In this study, the minimum threshold was selected at r = 0.3 (graph density ~ 60%) because below this level, group differences can be obscured by the inclusion of random, non-specific connections. Likewise, the maximum threshold was selected at r = 0.65 (graph density ~ 25%) because above this level, individual nodes may become disconnected from the rest of the graph and distort the results of group comparisons. For graph analysis, we applied the general linear model to the bootstrapped data obtained across connectivity thresholds, followed by post-hoc Bonferroni tests to evaluate differences in graph metrics between groups and timepoints. These analyses were performed using MATLAB R2023a, and the results were considered Significant for p < 0.05 (two-tailed), incorporating the Bonferroni correction for multiple comparisons.

#### Estimation of the time to phenoconversion based on network expression

To understand the relevance of the longitudinal imaging changes to clinical onset, we first determined whether high PDRP expression in iRBD is associated with greater likelihood of conversion^33^. To explore this possibility, we (R.B.P, J-F.G) obtained clinical follow-up on the 17 iRBD subjects (14 men and 3 women; age: 68.9 ± 4.8 years; baseline UPDRS motor ratings: 4.5 ± 3.3) from the Sleep Disorders Clinic of the Hôpital du Sacré-Coeur de Montréal (Montreal, Quebec, Canada) for whom baseline network imaging values were previously reported^33,49^. These participants underwent perfusion scanning with [^99m^Tc]-ethylcysteinate dimer (ECD) single photon emission computerized tomography (SPECT) at a single timepoint 12.8 ± 9.5 years from the diagnosis of iRBD, and were followed clinically for an additional 7.1 ± 5.2 years after imaging. In this observational cohort, 12 subjects (9 men and 3 women; age: 69.9 ± 5.1 years; baseline UPDRS motor ratings: 5.4 ± 2.8; iRBD duration: 9.6 ± 6.7 years) converted to either PD (n = 8) or DLB (n = 4) at an average of 4.8 ± 3.9 years after imaging. PDRP expression was computed in each of the scans, and the resulting values were z-scored with respect to analogous values from 17 age- and sex-matched HC subjects (HC3: 13 men and 4 women; age: 66.6 ± 6.0 years). Linear regression analysis was used to determine whether a Significant relationship existed between baseline PDRP expression and the time from imaging to phenoconversion. Multiple regression analysis and partial correlation leverage plots were used to assess the significance of this correlation after adjusting for individual differences in sex, age, duration of iRBD, and UPDRS motor ratings at the time of imaging. Correlations were considered Significant for p < 0.05.

#### Longitudinal analysis

We next examined the longitudinal imaging data in the current study to determine whether more accurate predictions were possible using PDRP expression in combination with caudate/putamen DAT binding measures. To this end, we made use of the progression rates for these measures estimated for the whole iRBD sample using IGM (see above) to predict the time to phenoconversion for each participant from the final pre-conversion imaging timepoint. The predicted time of phenoconversion was calculated as the number of years needed for *both* imaging measurements to reach published thresholds for early PD, i.e., PDRP expression ≥ + 1.5 (z-score) and putamen DAT binding ≤ 40% of normal mean^11,12,24^. The validity of this approach was explored in the members of iRBD cohort who phenoconverted during the clinical follow-up phase of the study. This was done by comparing the predicted time of phenoconversion based on the model to the time when phenoconversion actually took place. Statistical analyses were performed using SAS Studio (SAS Institute, Cary, NC), and all results were considered Significant at p < 0.05, two-tailed.

## Data Availability

Deidentified data will be made available on reasonable request from interested investigators for the purpose of replicating results.

## References

[1] Berg D. MDS research criteria for prodromal Parkinson’s disease. Mov. Disord. 30, 1600–1611 (2015). 26474317 10.1002/mds.26431

[2] Fernández-Arcos A., Iranzo A., Serradell M., Gaig C. & Santamaria J. The clinical phenotype of idiopathic rapid eye movement sleep behavior disorder at presentation: A study in 203 consecutive patients. Sleep 39, 121–132 (2016). 26940460 10.5665/sleep.5332 PMC4678361

[3] Galbiati A., Verga L., Giora E., Zucconi M. & Ferini-Strambi L. The risk of neurodegeneration in REM sleep behavior disorder: A systematic review and meta-analysis of longitudinal studies. Sleep Med. Rev. 43, 37–46 (2019). 30503716 10.1016/j.smrv.2018.09.008

[4] Postuma R. B. Risk and predictors of dementia and parkinsonism in idiopathic REM sleep behaviour disorder: A multicentre study. Brain 142, 744–759 (2019). 30789229 10.1093/brain/awz030 PMC6391615

[5] Schindlbeck K. A. & Eidelberg D. Network imaging biomarkers: insights and clinical applications in Parkinson’s disease. Lancet Neurol. 17, 629–640 (2018). 29914708 10.1016/S1474-4422(18)30169-8

[6] Rus T. Stereotyped Relationship Between Motor and Cognitive Metabolic Networks in Parkinson’s Disease. Mov. Disord. 37, 2247–2256 (2022). 36054380 10.1002/mds.29188 PMC9669200

[7] Perovnik M., Rus T., Schindlbeck K. A. & Eidelberg D. Functional brain networks in the evaluation of patients with neurodegenerative disorders. Nat. Rev. Neurol. 19, 73–90 (2023). 36539533 10.1038/s41582-022-00753-3

[8] Chahine L. M. Dopamine transporter imaging predicts clinically-defined α-synucleinopathy in REM sleep behavior disorder. Ann. Clin. Transl. Neurol. 8, 201–212 (2021). 33321002 10.1002/acn3.51269 PMC7818144

[9] Schindlbeck K. A. Cognition-Related Functional Topographies in Parkinson’s Disease: Localized Loss of the Ventral Default Mode Network. Cereb. Cortex 31, 5139–5150 (2021). 34148072 10.1093/cercor/bhab148 PMC8491681

[10] Schindlbeck K. A. LRRK2 and GBA Variants Exert Distinct Influences on Parkinson’s Disease-Specific Metabolic Networks. Cereb. Cortex 30, 2867–2878 (2020). 31813991 10.1093/cercor/bhz280 PMC7197067

[11] Huang C. Changes in network activity with the progression of Parkinson’s disease. Brain 130, 1834–1846 (2007). 17470495 10.1093/brain/awm086 PMC4454378

[12] Tang C. C., Poston K. L., Dhawan V. & Eidelberg D. Abnormalities in metabolic network activity precede the onset of motor symptoms in Parkinson’s disease. J. Neurosci. 30, 1049–1056 (2010). 20089913 10.1523/JNEUROSCI.4188-09.2010 PMC2866050

[13] Niethammer M. & Eidelberg D. Metabolic brain networks in translational neurology: Concepts and applications. Ann. Neurol. 72, 635–647 (2012). 22941893 10.1002/ana.23631 PMC4564117

[14] Ko J. H., Spetsieris P. G. & Eidelberg D. Network structure and function in Parkinson’s disease. Cereb. Cortex 28, 4121–4135 (2018). 29088324 10.1093/cercor/bhx267 PMC6215468

[15] Hawkes C. H., Del Tredici K. & Braak H. A timeline for Parkinson’s disease. Park. Relat. Disord. 16, 79–84 (2010). 10.1016/j.parkreldis.2009.08.007 19846332

[16] Newman M. E. J. Networks: An Introduction. (Oxford University Press, 2010).

[17] Noldus R. & Van Mieghem P. Assortativity in complex networks. J. Complex Networks 3, 507–542 (2015).

[18] Barabasi A.-L. Network Science. (Cambridge Univeristy Press, 2016).

[19] Murakami M., Ishikura S., Kominami D., Shimokawa T. & Murata M. Robustness and efficiency in interconnected networks with changes in network assortativity. Appl. Netw. Sci. 2, 6 (2017). 30533514 10.1007/s41109-017-0025-4 PMC6245120

[20] Peel L., Delvenne J. C. & Lambiotte R. Multiscale mixing patterns in networks. Proc. Natl. Acad. Sci U. S. A. 115, 4057–4062 (2018). 29610344 10.1073/pnas.1713019115 PMC5910813

[21] Reisinger D., Adam R., Tschofenig F., Füllsack M. & Jäger G. Modular tipping points: How local network structure impacts critical transitions in networked spin systems. PLoS One 18, e0292935 (2023). 37963138 10.1371/journal.pone.0292935 PMC10645300

[22] Vo A. Adaptive and pathological connectivity responses in Parkinson’s disease brain networks. Cereb. Cortex 33, 917–932 (2023). 35325051 10.1093/cercor/bhac110 PMC9930629

[23] Iranzo A. Serial dopamine transporter imaging of nigrostriatal function in patients with idiopathic rapid-eye-movement sleep behaviour disorder: A prospective study. Lancet Neurol. 10, 797–805 (2011). 21802993 10.1016/S1474-4422(11)70152-1

[24] Tang C. C. Hemispheric Network Expression in Parkinson’s Disease: Relationship to Dopaminergic Asymmetries. J. Parkinsons. Dis. 10, 1737–1749 (2020). 32925097 10.3233/JPD-202117

[25] Carli G. Occipital hypometabolism is a risk factor for conversion to Parkinson’s disease in isolated REM sleep behaviour disorder. Eur. J. Nucl. Med. Mol. Imaging 50, 3290–3301 (2023). 37310428 10.1007/s00259-023-06289-y PMC10542098

[26] Arnaldi D. Clinical and dopaminergic imaging characteristics of the FARPRESTO cohort of trial-ready idiopathic rapid eye movement sleep behavior patients. Eur. J. Neurol. 30, 3703–3710 (2023). 37498611 10.1111/ene.16001

[27] Wu P. Consistent abnormalities in metabolic network activity in idiopathic rapid eye movement sleep behaviour disorder. Brain 137, 3122–3128 (2014). 25338949 10.1093/brain/awu290 PMC4240297

[28] Meles S. K. The Metabolic Pattern of Idiopathic REM Sleep Behavior Disorder Reflects Early-Stage Parkinson Disease. J. Nucl. Med. 59, 1437–1444 (2018). 29476004 10.2967/jnumed.117.202242

[29] Kim R. Longitudinal Changes in Isolated Rapid Eye Movement Sleep Behavior Disorder-Related Metabolic Pattern Expression. Mov. Disord. 36, 1889–1898 (2021). 33788284 10.1002/mds.28592 PMC8451853

[30] Shin J. H. Parkinson Disease-Related Brain Metabolic Patterns and Neurodegeneration in Isolated REM Sleep Behavior Disorder. Neurology 97, e378–e388 (2021). 34011571 10.1212/WNL.0000000000012228

[31] Rahayel S. 99mTc-HMPAO SPECT Perfusion Signatures Associated With Clinical Progression in Patients With Isolated REM Sleep Behavior Disorder. Neurology 102, e208015 (2024). 38315966 10.1212/WNL.0000000000208015 PMC10890831

[32] Feigin A. Tc-99m ethylene cysteinate dimer SPECT in the differential diagnosis of parkinsonism. Movement Disorders vol. 17 1265–1270 (2002). 12465066 10.1002/mds.10270

[33] Holtbernd F. Abnormal metabolic network activity in REM sleep behavior disorder. Neurology 82, 620–627 (2014). 24453082 10.1212/WNL.0000000000000130 PMC3963420

[34] Peng S. Dynamic 18F-FPCIT PET: Quantification of Parkinson Disease Metabolic Networks and Nigrostriatal Dopaminergic Dysfunction in a Single Imaging Session. J. Nucl. Med. 62, 1775–1782 (2021). 33741649 10.2967/jnumed.120.257345 PMC8612203

[35] Vo A. Parkinson’s disease-related network topographies characterized with resting state functional MRI. Hum. Brain Mapp. 38, 617–630 (2017). 27207613 10.1002/hbm.23260 PMC5118197

[36] Rommal A. Parkinson’s disease-related pattern (PDRP) identified using resting-state functional MRI: Validation study. Neuroimage: Reports 1, 100026 (2021). 10.1016/j.ynirp.2021.100026 PMC12172760 40567285

[37] Niethammer M. Gene therapy reduces Parkinson’s disease symptoms by reorganizing functional brain connectivity. Sci. Transl. Med. 10, eaau0713 (2018). 30487248 10.1126/scitranslmed.aau0713

[38] Spetsieris P. G. & Eidelberg D. Scaled subprofile modeling of resting state imaging data in Parkinson’s disease: Methodological issues. Neuroimage 54, 2899–2914 (2011). 20969965 10.1016/j.neuroimage.2010.10.025 PMC3020239

[39] Niethammer M. Long-term follow-up of a randomized AAV2-GAD gene therapy trial for Parkinson’s disease. JCI Insight 2, e90133 (2017). 28405611 10.1172/jci.insight.90133 PMC5374069

[40] Niethammer M. A Network Imaging Biomarker of X-Linked Dystonia-Parkinsonism. Ann. Neurol. 94, 684–695 (2023). 37376770 10.1002/ana.26732

[41] Tang C. C. Metabolic network as a progression biomarker of premanifest Huntington’s disease. J. Clin. Invest. 123, 4076–4088 (2013). 23985564 10.1172/JCI69411 PMC3754266

[42] Ghisletta P. On the use of growth models to study normal cognitive aging. Int. J. Behav. Dev. 44, 88–96 (2020).

[43] Tzourio-Mazoyer N. Automated anatomical labeling of activations in SPM using a macroscopic anatomical parcellation of the MNI MRI single-subject brain. Neuroimage 15, 273–289 (2002). 11771995 10.1006/nimg.2001.0978

[44] Vo A. Disordered network structure and function in dystonia: pathological connectivity vs. adaptive responses. Cereb. Cortex 33, 6943–6958 (2023). 36749014 10.1093/cercor/bhad012 PMC10233302

[45] Rubinov M. & Sporns O. Complex network measures of brain connectivity: Uses and interpretations. Neuroimage 52, 1059–1069 (2010). 19819337 10.1016/j.neuroimage.2009.10.003

[46] Bassett D. S. & Bullmore E. T. Small-World Brain Networks Revisited. Neuroscientist 23, 499–516 (2017). 27655008 10.1177/1073858416667720 PMC5603984

[47] Wu X. Z., Fennell P. G., Percus A. G. & Lerman K. Degree correlations amplify the growth of cascades in networks. Phys. Rev. E 98, 022321 (2018). 30253536 10.1103/PhysRevE.98.022321

[48] Alexander B., Pushkar A. & Girvan M. Phase transitions and assortativity in models of gene regulatory networks evolved under different selection processes. J. R. Soc. Interface 18, 20200790 (2021). 10.1098/rsif.2020.0790 PMC8086935 33849335

[49] Dang-Vu T. T. Hippocampal perfusion predicts impending neurodegeneration in REM sleep behavior disorder. Neurology 79, 2302–2306 (2012). 23115214 10.1212/WNL.0b013e318278b658 PMC3578380

